# Clinical characteristics and blood/serum bound prognostic biomarkers in advanced pancreatic cancer treated with gemcitabine and nab-paclitaxel

**DOI:** 10.1186/s12885-020-07426-8

**Published:** 2020-10-02

**Authors:** Hakon Blomstrand, Henrik Green, Mats Fredrikson, Emma Gränsmark, Bergthor Björnsson, Nils O. Elander

**Affiliations:** 1 Department of Clinical Pathology and Department of Biomedical and Clinical Sciences, Linköping University, 58183 Linköping, Sweden; 2 Division of Drug Research, Department of Medical Health Sciences, Linköping University, 58183 Linköping, Sweden; 3 Department of Forensic Genetics and Forensic Toxicology, National Board of Forensic Medicine, 58758 Linköping, Sweden; 4 Forum Östergötland, Linköping University, 58185 Linköping, Sweden; 5 Department of Oncology, Kalmar County Hospital, 392 44 Kalmar, Sweden; 6 Department of Surgery and Department of Biomedical and Clinical Sciences, Linköping University, 58183 Linköping, Sweden; 7 Department of Oncology and Department of Biomedical and Clinical Sciences, Linköping University, 58183 Linköping, Sweden

**Keywords:** Pancreatic cancer, Gemcitabine, Nab-paclitaxel, Prognostic markers, Serum albumin

## Abstract

**Background:**

In recent years treatment options for advanced pancreatic cancer have markedly improved, and a combination regimen of gemcitabine and nab-paclitaxel is now considered standard of care in Sweden and elsewhere. Nevertheless, a majority of patients do not respond to treatment. In order to guide the individual patient to the most beneficial therapeutic strategy, simple and easily available prognostic and predictive markers are needed.

**Methods:**

The potential prognostic value of a range of blood/serum parameters, patient-, and tumour characteristics was explored in a retrospective cohort of 75 patients treated with gemcitabine/nab-paclitaxel (Gem/NabP) for advanced pancreatic ductal adenocarcinoma (PDAC) in the South Eastern Region of Sweden. Primary outcome was overall survival (OS) while progression free survival (PFS) was the key secondary outcome.

**Result:**

Univariable Cox regression analysis revealed that high baseline serum albumin (> 37 g/L) and older age (> 65) were positive prognostic markers for OS, and in multivariable regression analysis both parameters were confirmed to be independent prognostic variables (HR 0.48, *p* = 0.023 and HR = 0.47, *p* = 0.039,). Thrombocytopenia at any time during the treatment was an independent predictor for improved progression free survival (PFS) but not for OS (HR 0.49, *p* = 0.029, 0.54, *p* = 0.073), whereas thrombocytopenia developed under cycle 1 was neither related with OS nor PFS (HR 0.87, *p* = 0.384, HR 1.04, *p* = 0.771). Other parameters assessed (gender, tumour stage, ECOG performance status, myelosuppression, baseline serum CA19–9, and baseline serum bilirubin levels) were not significantly associated with survival.

**Conclusion:**

Serum albumin at baseline is a prognostic factor with palliative Gem/NabP in advanced PDAC, and should be further assessed as a tool for risk stratification. Older age was associated with improved survival, which encourages further studies on the use of Gem/NabP in the elderly.

## Background

Pancreatic ductal adenocarcinoma (PDAC) is a malignancy with poor prognosis and one of the top five reasons for cancer-related death in both Europe and North America. Although therapeutic options have improved, it is still one of few cancers with increasing mortality [1]. The majority of PDAC patients have disseminated disease already at diagnosis with tumour specific treatment options restricted to palliative chemotherapy. For almost 20 years, gold standard treatment was single agent chemotherapy with gemcitabine based on the milestone phase III trial published by Burris et al in 1997 [2]. More recently, improved survival has been achieved with different combination chemotherapy regimens such as FOLFIRINOX [3] and gemcitabine/nab-paclitaxel (Gem/NabP) [4]. Due to its safety and tolerability profile, and since the combination was approved by the regulatory authorities in 2014, many centres in Sweden have preferred Gem/NabP as first line palliative treatment of advanced PDAC.

However, with many patients still not responding well to treatment, selecting the right patient for chemotherapy (and the most suitable type of chemotherapy) is essential. Unfortunately clinically validated predictive markers for chemotherapy treatments used in advanced PDAC are lacking. Prognostic markers are also sparse, although there is some evidence of prognostic value in biomarkers such as CA19–9 [5]. While several assays for molecular profiling and treatment stratification of PDAC have been proposed (reviewed in [6]), none of these attempts have so far reached routine clinical practice. Thus the need for simple, easily available, and clinically relevant prognostic parameters remains high. In the present study, a panel of routine serum parameters such as CA19–9 and albumin as well as standard blood parameters (haemoglobin, white blood cell count, neutrophils, and platelets) were analysed in 75 real world patients with advanced PDAC treated with Gem/NabP. The potential prognostic value of the blood/serum biomarkers was explored in terms of the primary endpoint overall survival (OS) as well as the secondary endpoint progression free survival (PFS). Similarly, the prognostic value of baseline clinical characteristics including age, gender, ECOG performance status, and disease burden, was assessed.

## Methods

### Patients

As previously presented in this journal, a retrospective observational study covering the 75 first patients treated with first line Gem/NabP due to locally advanced or metastatic PDAC was conducted in the South East region of Sweden [7]. Inclusion/exclusion criteria, patient and treatment characteristics, and data collection are extensively described in this earlier publication [7].

### Blood and serum analyses

All relevant information on blood and serum analyses was manually extracted from medical records. Serum CA19–9, albumin, and bilirubin, as well as blood counts (haemoglobin, white blood cell counts, neutrophils, and platelets) were analysed according to clinical routine at accredited laboratories at the respective hospital (Kalmar County Hospital, Linköping University Hospital and Ryhov County hospital in Jönköping). Due to site-specific differences in blood sampling, comparative analyses of factors such as lactate dehydrogenase (LDH), CRP and neutrophil-lymphocyte-ratio (NLR) were not possible. Bone marrow toxicity, i.e. myelosuppression and thrombocytopenia, was graded according to National Cancer Institute Common Terminology Criteria for Adverse Events (CTCAE), version 4.

### Statistical analysis

All patients receiving at least one dose of Gem/NabP for treatment of advanced PDAC were included in the analysis. Median overall survival (OS) and median progression free survival (PFS) in subgroups based on relevant clinical and biochemical parameters were estimated using Kaplan-Meier survival analyses and compared using the log rank test. The primary endpoint OS was defined as time from start of treatment until date of death or last follow-up. For S-albumin the mean value of 37 g/l, which is very close to the commonly used lower normal limit (36 g/L), was used to dichotomise the cohort into a ‘low’ and ‘high’ group and further used in the univariable analyses. Regarding CA19–9, there is no universally accepted cut-off value. In this study the median value was used to dichotomise the material into a ‘low’ and ‘high’ group as well as the value of 59 times the upper normal limit (ULN), which was used in a previous phase III study [4]. A *p*-value of less than 0.15 in univariable analysis was considered relevant for inclusion in multivariable analysis. A Cox proportional hazards regression model was applied with relevant parameters from univariable analysis as well as established risk factors, i.e. ECOG performance status and disease stage (locally advanced or metastatic disease), to determine independent prognostic factors. Multivariable analysis was stratified for treatment length (≤3 or > 3 treatment cycles) to minimise bias from haematotoxicity parameters not being time specific.

Secondary endpoint (PFS) was defined as time from start of treatment until progression, either radiological or clinical, or death, whatever came first. The same biochemical and clinical parameters analysed in univariable- and multivariable OS analysis were also applied to the PFS data. Student’s t-test was utilized to compare treatment length among patients with and without thrombocytopenia during the treatment course. In the further analyses of patient characteristics in the subgroups according to age >/≤65 years *p*-values for comparisons between quota, mean, and median values were calculated with chi2-test, t-test, and Mann-Whitney test, respectively. SPSS v24 (IBM Corp. Armonk NY) and Statistica v13.2 (Dell, Inc. Round Rock, TX) were used for statistical analyses.

## Results

As previously described, a cohort of 75 patients was identified. PDAC diagnosis was based on histology/cytology in 63 patients (84%) and radiology with or without serum markers in 12 (16%). Basic patient characteristics, treatment parameters and safety, as well as survival data in the overall population, have been published previously [7]. A summary of survival data in the total population and subpopulations is presented in Tables 1 and 2.

**Table 1:** Univariable and multivariable analyses for OS data

	mOS	HR univariable (95% CI)		*p-*value	HR multivariable (95% CI)	*p*-value
Entire cohort (95% CI)	10.9 (7.8–14.0)					
Age ≤ 65	6.9					
Age > 65	13.2	0.57 (0.32–1.02)		0.062	0.50 (0.26–0.96)	**0.039**
Locally advanced	17.1					
Metastasized	9.4	1.62 (0.85–3.09)		0.114	1.45 (0.73–2.94)	0.287
ECOG PS 0	14.5					
ECOG PS 1–2	9.4	1.37 (0.76–2.48)		0.288	1.16 (0.63–2.17)	0.633
Ca19–9 ≤ median^a^	11.0					
Ca19–9 > median	10.4	1.12 (0.61–2.07)		0.715		
Ca19–9 < 59xULN	11.3					
Ca19–9 ≥ 59xULN	6.8	1.59 (0.84–3.01)		0.187		
Albumin ≤37 g/L	8.3					
Albumin > 37 g/L	14.8	0.53 (0.30–0.95)		**0.032**	0.48 (0.26–0.90)	**0.023**
TPK grade 0	6.8					
TPK grade 1–4	14.7	0.29 (0.16–0.55)		**0.001**	0.54 (0.27–1.06)	0.073
Normal bilirubin^b^	11.3					
Elevated bilirubin	10.1	1.59 (0.56–4.51)		0.421		
Dose reduction	10.9					
Full dose	6.2	1.77 (0.90–3.51)		0.119	1.03 (0.50–2.13)	0.942
No 2nd line	8.2					
2nd line	12.0	0.80 (0.45–1.42)		0.429		
BM-tox grade 0–1	7.0					
BM-tox grade 2–4	14.5	0.46 (0.25–0.84)		**0.017**		
BM-tox grade 0–2	8.9					
BM-tox grade 3–4	not reached^c^	0.41 (0.20–0.87)		**0.012**	0.58 (0.26–1.27)	0.172
No leucocytosis^d^	11.9					
Leucocytosis	8.2	1.41 (0.78–2.57)		0.272		

Median OS in months. Abbreviations: *HR* Hazard ratio, *CI* Confidence interval, *PS* Performance status, *TPK* Platelet count, *BM* Bone marrow, *LPK* White blood cell count, *ULN* Upper limit of normal. ^a^Median CA19–9 was 567kU/l, ^b^S-bil < 26 μmole/L at treatment start, ^c^ Due to censored cases (61%),^d^ < 8.8 × 10^9^/L at treatment start. Statistical significance at the 0.05 level marked in bold text.

**Table 2:** Univariable and multivariable analyses for PFS data

	mPFS	HR univariable (95% CI)		*p*-value	HR multivariable (95% CI)	*p*-value
Entire cohort (95% CI)	5.2 (3.4–7.0)					
Age ≤ 65	3.3					
Age > 65	7.7	0.66 (0.40–1.09)		0.113	0.64 (0.37–1.10)	0.108
Locally advanced	6.8					
Metastasized	4.5	1.22 (0.72–2.09)		0.434	1.55 (0.80–2.98)	0.190
ECOG PS 0	6.2					
ECOG PS 1–2	4.5	1.08 (0.65–1.79)		0.769	1.16 (0.68–2.00)	0.579
Ca19–9 < median^a^	5.4					
Ca19–9 > median	5.1	1.15 (0.68–1.96)		0.597		
Ca19–9 < 59xULN	4.2					
Ca19–9 ≥ 59xULN	6.4	1.43 (0.82–2.49)		0.228		
Albumin ≤37	5.1					
Albumin > 37	6.1	0.72 (0.44–1.19)		0.194	0.93 (0.53–1.67)	0.826
TPK grade 0	3.1					
TPK grade 1–4	7.0	0.31 (0.18–0.55)		**< 0.001**	0.49 (0.26–0.93)	**0.029**
Normal bilirubin^b^	5.2					
Elevated bilirubin	4.0	1.56 (0.56–4.34)		0.482		
Dose reduction	6.4					
Full dose	3.0	2.10 (1.13–3.93)		**0.045**	1.15 (0.57–2.33)	0.698
BM-tox grade 0–1	4.2					
BM-tox grade 2–4	6.8	0.50 (0.29–0.85)		**0.021**		
BM-tox grade 0–2	5.1					
BM-tox grade 3–4	6.2	0.41 (0.20–0.87)		0.137	0.80 (0.41–1.56)	0.506
No leucocytosis^c^	4.5					
Leucocytosis	6.5	0.96 (0.57–1.63)		0.886		

Median PFS in months. ^a^Median CA19–9 was 567kU/l, ^b^S-bil < 26 μmole/L at treatment start, ^c^ < 8.8 × 10^9^/L at treatment start

**Fig. 1:**
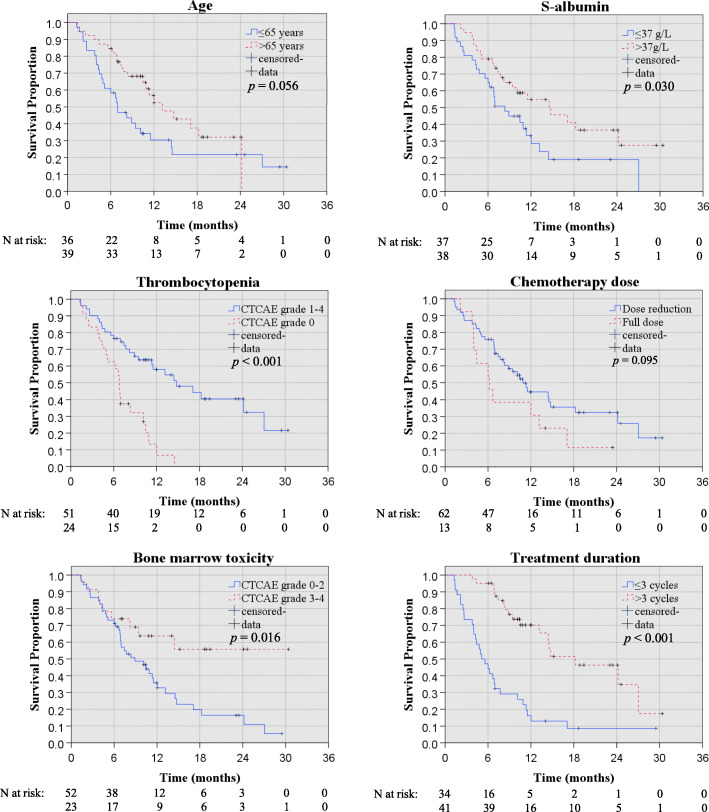
Kaplan Meier survival plots for parameters included in OS multivariable analysis with *p*-values for log rank test

**Fig. 2:**
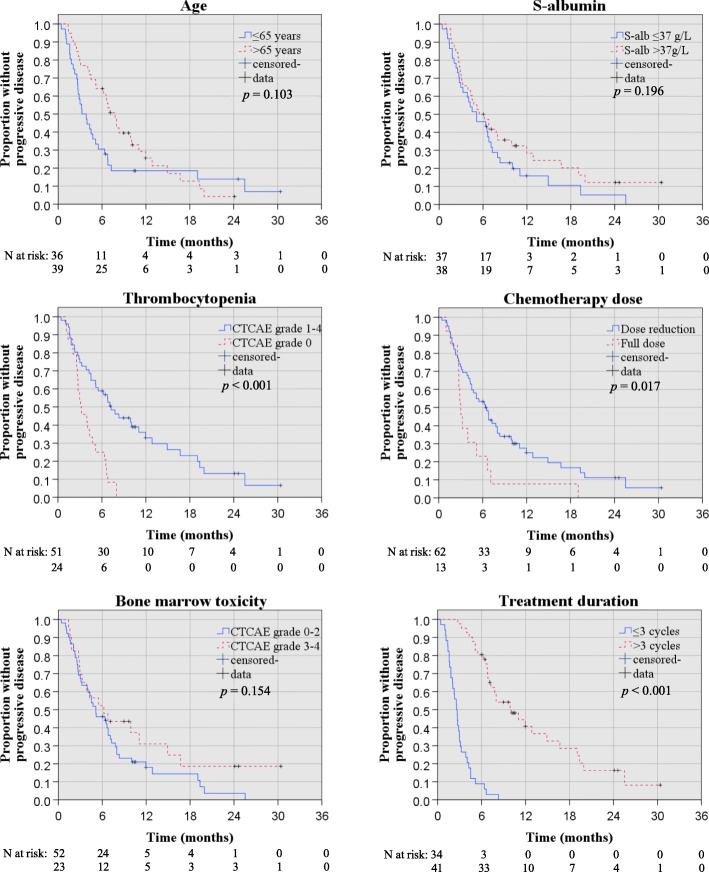
Kaplan Meier survival plots for parameters included in PFS multivariable analysis, with *p*-values for log rank test

OS and PFS estimates for subgroups according to age, gender, ECOG performance status, tumour stage, serum CA19–9, serum albumin, serum bilirubin, myelosuppression, and occurrence of dose reduction, were made with Kaplan Meier survival analyses as seen in Figs. 1 and 2.

### Univariable regression analyses

The results of the univariable Cox regression analyses with hazard ratios (HR) for OS and PFS are displayed in Tables 1 and 2, revealing that serum albumin before treatment start (cut off 37 g/l, HR 0.53, *p* = 0.032), occurrence of thrombocytopenia (all CTCAE grades during treatment, HR 0.29, *p* = 0.001), and bone marrow toxicity (CTCAE grade 3–4 during treatment, HR 0.41, *p* = 0.012) were statistically significant prognostic markers predicting death (Table 1). A statistical trend, although not significant, was found between age (inverted correlation, HR 0.57, *p* = 0.062) and OS.

Similarly, with regard to secondary endpoint PFS, univariable analyses revealed that the occurrence of thrombocytopenia of any CTCAE grade during treatment (HR 0.31, *p =* < 0.001), treatment dose (HR 2.10, *p* = 0.045), and bone marrow toxicity (CTCAE grade 2–4 during treatment, HR 0.50, *p* = 0.021) were statistically significant for the prediction of progressive disease (Table 2).

### Multivariable regression analyses

Parameters considered statistically relevant with regard to OS were further analysed in multivariable regression analyses; age >/≤ 65 years (inverted, with older age being associated with improved survival, HR 0.50, *p* = 0.039), and S-Albumin (cut off 37 g/l, HR 0.48, *p* = 0.023), were statistically significant with regard to the prediction of death (Table 1). A statistical trend, although not significant, was found between occurrence of thrombocytopenia (all CTCAE grades, HR 0.54, *p* = 0.073) and OS.

Multivariable regression analyses with regard to the secondary endpoint PFS confirmed that thrombocytopenia of any CTCAE grade (HR 0.49, *p* = 0.029) was an independent prognostic marker for progression (Table 2). A statistical trend, although not significant, was found between age (HR 0.62, *p* = 0.108, with older age related to lower risk) and PFS. The inclusion of covariates in terms of occurrence of second line therapy and bone marrow toxicity of CTCAE grade 2–4 (instead of grade 3–4) did not affect the outcome of the multivariable analyses (data not shown).

### Baseline characteristics and treatment data in ‘old’ vs. ‘young’ patients

As the finding of age above 65 years being a positive prognostic factor for OS was somewhat unexpected, further analyses of the characteristics of the age subgroups were made. While no statistically significant differences concerning baseline characteristics were evident in old and young patients, there was a trend towards more patients with metastatic disease (81% vs 62%, *p* = 0.070), higher median CA19–9 (980 vs 475.5, *p* = 0.057), and a history of less previous adjuvant chemotherapy (22% vs 41%, *p* = 0.081) in patients ≤65. Dose intensity of NabP, but not Gem, was significantly lower in the ‘old’ subgroup (63% vs 75%, *p* = 0.028, Table 3.)

**Table 3:** Patient characteristics in subgroups according to age >/≤ 65 years

	≤65 (*n* = 36)	> 65 (*n* = 39)	*p-*value
Female	18 (50)	16 (41)	0.435
Prior Surgery	10 (28)	17 (44)	0.154
Localised disease/M0	7 (19)	15 (38)	0.070
CA19–9(median)^a^	980	476	0.057
ECOG 0	17 (47)	16 (41)	0.589
ECOG 1	17 (47)	19 (49)	0.897
ECOG 2	2 (6)	4 (10)	0.453
Adjuvant chemotherapy	8 (22)	16 (41)	0.081
Neoadjuvant chemotherapy	2 (6)	1 (3)	0.509
S-albumin (mean)	37.7	36.6	0.335
S-albumin (median)	39	37	0.203
N cycles (median)	3	5	0.061
Thrombocytopenia	21 (58)	27 (69	0.326
BM-toxicity G3–4	9 (25)	14 (36)	0.307
Dose intensity Gem (mean)	79%	73%	0.214
Dose intensity NabP (mean)	75%	63%	**0.028**
Full dose	25%	10%	0.092
2nd line treatment^b^	45%	55%	0.438

Number (%) where not otherwise stated. ^a^Dropouts were 5 and 3 in the young and elderly group, respectively. ^b^Dropouts were 5 and 10 in the young and elderly group, respectively. Statistical significance at the 0.05 level marked in bold text.

### Comparisons in patients with and without treatment associated thrombocytopenia

Further analyses were conducted in the subgroups of patients with and without signs of thrombocytopenia during the treatment course. Student’s t-test confirmed longer treatment duration in patients where thrombocytopenia occurred, with average number of treatment cycles of 6.3 and 3.4 in the thrombocytopenia versus the non-thrombocytopenia groups, respectively (*p* = 0.003) (Table 4).

**Table 4:** T-test showing mean treatment duration (number of treatment cycles) for patients with or without thrombocytopenia reported

	No thrombocytopenia	Thrombocytopenia	t-value	*p*-value
Cycles (n)	3.4	6.3	−3.0	0.003
SD	1.79	4.52		

Abbreviations: *SD* Standard deviation

**Fig. 3:**
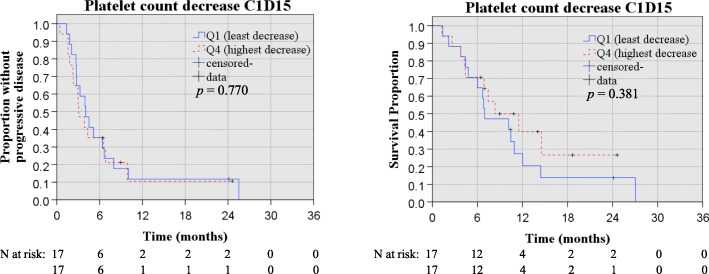
Kaplan Meier curve estimating OS and PFS for quartiles with highest and lowest platelet toxicity first treatment cycle with *p*-value for log rank test

In addition, overall survival in the quartile of patients with the most pronounced reduction of blood platelet count at cycle 1 day 15 was compared with the quartile of patients with the least pronounced (or no) reduction of platelets at this time point, revealing no significant difference (Fig. 3).

## Discussion

This study provides real world data on baseline clinical characteristics and blood/serum based biomarkers of prognostic significance in patients with advanced PDAC treated with Gem/NabP. As previously described [7], the efficacy and safety of the Gem/NabP regimen seems comparable in real world and the randomised controlled trial context. While some patients clearly benefit from this treatment, others do not, and a substantial part of the patients will experience rapid progression and death. Hence it is essential to define prognostic and predictive parameters, in order to select the right patient to the right type of treatment.

In the present study, multivariable regression analyses revealed that age > 65 years and baseline serum albumin > 37 g/L were independent prognostic markers with regard to overall survival, whereas the occurrence of thrombocytopenia (of any CTCAE grade ≥ 1) was an independent marker for PFS but not for OS.

Notably, median overall survival was almost doubled in elderly vs. younger patients (13.2 vs 6.9 months in patients >/≤ 65 years, HR 0.5, *p* = 0.039). This was similarly reflected in terms of PFS, although the difference did not reach statistical significance in multivariable analysis. As this was an unexpected finding, patient, tumour, and treatment characteristics in the two respective groups were explored more thoroughly. The only significant difference found between the groups was the dose intensity of nab-paclitaxel, which was slightly lower in the elderly group (Table 3). While there was a tendency towards lower proportion of metastasised patients, higher baseline CA19–9 levels, and higher proportion of prior adjuvant treatment in the ‘old’ subgroup, it remains to be elucidated whether age per se is a prognostic factor or not. Notably, and in contrast to our results, subgroup analyses in the MPACT phase III trial cohort [4] and long-term follow-up [5] indicated that survival was *worse* among patients older than 65 years. On the other hand, a retrospective study on Japanese patients reported similar survival data in patients above and below 75 years age [8]. While the differences observed may be due to different study populations and inclusion/exclusion criteria, e.g. the inclusion or exclusion of patients with locally advanced or relapsing PDAC, the combined evidence implies that further prospective studies focusing on optimal treatment protocols for ‘old’ and ‘young’ patients with pancreatic cancer are relevant. A currently recruiting phase IV trial by Betke and co-workers [9] is investigating the outcome and safety in elderly pancreatic cancer patients guided to either Gem/NabP, Gem monotherapy, or best supportive care without chemotherapy, depending on the general fitness/frailty of the patient, based on a geriatric scoring model.

With regard to the value of serum albumin at baseline as a prognostic factor, little has been previously known in the palliative context. However, the present data are consistent with previous reports on long term survival in patients with earlier stages of pancreatic cancer who underwent pancreatic resection [10]. Serum albumin is often considered a surrogate marker for nutritional status, ‘general fitness’, and ability to recover following major abdominal surgery, and has been assessed in numerous trials on resectable pancreatic cancer (reviewed in [11]). The present study implies that serum albumin may be additionally useful as a prognostic marker in the palliative setting, in terms of predicting survival in patients commencing first line Gem/NabP palliative chemotherapy. Nevertheless, and as high serum albumin may be associated with enhanced risk of developing neutropenia in this type of patients treated with Gem/NabP [12], close monitoring of adverse events and tolerability is recommended in order to adjust the dosage and treatment schedules.

The present study further assessed whether standard bone marrow parameters in general could be used to predict survival under and following first line Gem/NabP chemotherapy in advanced pancreatic cancer. Thrombocytopenia, of any grade and at any time during the treatment course, was significantly associated with improved progression free survival. Individuals where thrombocytopenia was reported displayed more than doubled estimates on PFS (7.0 vs 3.1, HR multivariable 0.49, *p* = 0.029) and a trend towards improved OS was similarly observed (14.7 vs 6.8 months, HR multivariable 0.54, *p* = 0.073). Based on this result it is tempting to speculate that treatment induced thrombocytopenia could be a potential treatment predictive biomarker, but the finding that patients who experienced thrombocytopenia also had significantly longer duration of treatment (6.3 vs 3.4 cycles, *p* = 0.003) raises concern regarding the validity of such a conclusion. Naturally, patients with longer treatment duration are also exposed to greater treatment related risks. In addition, if the development of thrombocytopenia would be of any clinical relevance in terms treatment planning it must, self-evidently, occur early and not late in the treatment course.

To dissect this in more detail, we therefore explored whether early onset of thrombocytopenia (under cycle 1) was a predictive factor for the long term benefit of the Gem/NabP treatment (Fig. 3). The latter analysis did not reveal any significant survival differences between patients with and without early onset thrombocytopenia, which indicates that the potential value of using thrombocytopenia as a clinically relevant predictor for treatment success is limited, at least in this situation.

Baseline serum levels of CA19–9, perhaps the most established pancreatic cancer ‘specific’ serum biomarker in clinical use, did not relate to overall or progression free survival in the present patient cohort. Since there is no standardised cut off for high/low (apart from the upper limit of normal), the median value (567 kU/L) was chosen to dichotomise the cohort into high versus low. Taberno et al [13], who similarly failed to reveal a prognostic impact of CA19–9 in the MPACT phase III population, utilised 59 times ULN as cut off. In a complementing analysis, we applied the same cut off as Taberno, with similar result i.e. no significant differences between CA19–9 high and low patients, respectively.

The present study has some limitations. The retrospective setup and lack of control population makes firm conclusions about specific drug – disease effects difficult. However, it does provide key data about prognostic parameters in a population of real world patients, including many individuals that would probably not meet the strict inclusion/exclusion criteria usually aligned to a randomised controlled trial. Together with previous phase III data, the present results may therefore aid in clinical decision making in the clinical reality of unselected patients treated outside the frame of a controlled trial.

## Conclusion

The present study provides real world evidence on clinical characteristics and blood/serum based prognostic markers in patients with advanced PDAC treated with Gem/NabP. High serum albumin at baseline was associated with improved survival, and should be further explored as a potential tool for risk stratification. In addition, age above 65 years was found to be an independent positive prognostic factor for survival, encouraging further studies on the use of Gem/NabP in elder patients with advanced PDAC. Other baseline analyses, including serum CA19–9, bilirubin, and early development of thrombocytopenia, did not predict survival in this patient cohort.

## Data Availability

The datasets used in the current study are available from the corresponding author on request.

## Abbreviations

PDAC: Advanced pancreatic ductal adenocarcinoma
Gem/NabP: Gemcitabine and nab-paclitaxel
NLR: Neutrophil-to-lymphocyte ratio
CTCAE: National Cancer Institute Common Terminology Criteria for Adverse Events
mOS: Median Overall Survival
mPFS: Median Progression Free Survival
ECOG: Eastern Cooperative Oncology Group
ULN: Upper limit of normal
HR: Hazard ratio
